# Targeting lipid metabolism as a new therapeutic strategy for inherited cardiomyopathies

**DOI:** 10.3389/fcvm.2023.1114459

**Published:** 2023-01-19

**Authors:** Karen R. Gaar-Humphreys, Alyssa van den Brink, Mark Wekking, Folkert W. Asselbergs, Frank G. van Steenbeek, Magdalena Harakalova, Jiayi Pei

**Affiliations:** ^1^Division Heart and Lungs, Department of Cardiology, Circulatory Health Research Center, University Medical Center Utrecht, Utrecht University, Utrecht, Netherlands; ^2^Regenerative Medicine Center Utrecht, University Medical Center Utrecht, Utrecht, Netherlands; ^3^Department of Cardiology, Amsterdam University Medical Centers, University of Amsterdam, Amsterdam, Netherlands; ^4^Health Data Research United Kingdom and Institute of Health Informatics, University College London, London, United Kingdom; ^5^Department of Clinical Sciences, Faculty of Veterinary Medicine, Utrecht University, Utrecht, Netherlands; ^6^Netherlands Heart Institute, Utrecht, Netherlands

**Keywords:** hypertrophic cardiomyopathy, dilated cardiomyopathy, genetic variants, lipid metabolism, fatty acid oxidation, transcription factor PPARA

## Abstract

Inherited cardiomyopathies caused by pathological genetic variants include multiple subtypes of heart disease. Advances in next-generation sequencing (NGS) techniques have allowed for the identification of numerous genetic variants as pathological variants. However, the disease penetrance varies among mutated genes. Some can be associated with more than one disease subtype, leading to a complex genotype-phenotype relationship in inherited cardiomyopathies. Previous studies have demonstrated disrupted metabolism in inherited cardiomyopathies and the importance of metabolic adaptations in disease onset and progression. In addition, genotype- and phenotype-specific metabolic alterations, especially in lipid metabolism, have been revealed. In this mini-review, we describe the metabolic changes that are associated with dilated cardiomyopathy (DCM) and hypertrophic cardiomyopathy (HCM), which account for the largest proportion of inherited cardiomyopathies. We also summarize the affected expression of genes involved in fatty acid oxidation (FAO) in DCM and HCM, highlighting the potential of PPARA-targeting drugs as FAO modulators in treating patients with inherited cardiomyopathies.

## Introduction

Inherited cardiomyopathies are diseases of the heart muscle due to pathological genetic variants. Based on the clinical presentations, they are often divided into four subtypes, namely, hypertrophic cardiomyopathy (HCM), dilated cardiomyopathy (DCM), arrhythmogenic cardiomyopathy (ACM), and restrictive cardiomyopathy (RCM) (1, 2). Among them, the estimated prevalence of DCM is the highest (1:250 individuals), followed by HCM (1:500 individuals) (3). Owing to the advances in next-generation sequencing (NGS) techniques, numerous genetic variants have been identified as disease-causing variants. To date, more than 60 mutated genes are associated with DCM, and they are involved in a wide range of cellular features, including the sarcomere, Z disk, cytoskeleton, sarcoplasmic reticulum and cytoplasm, ion channels, and mitochondria (4). A subset of those mutated genes, such as *TTN*, *LMNA*, *MYH7*, and *PLN*, exhibits a stronger gene-disease relationship with DCM compared to the rest (5). Unlike the broad range of DCM-causal variants, most pathological variants in HCM affect sarcomeric genes (6). *MYH7* and *MYBPC3* are the most commonly affected genes in HCM, which account for about 70% of those variants (7). The disease penetrance varies among mutated genes, and the same mutated gene can be associated with more than one subtype (2, 8), leading to a complex genotype-phenotype relationship in inherited cardiomyopathies. Therefore, molecular insights into the affected pathways and biological processes concerning different variants and/or subtypes are needed to characterize the diseases better and provide druggable candidates for novel treatments.

## Metabolic changes in inherited cardiomyopathies

The heart has a very high energy demand to fulfill its basic functions. Therefore, sufficient cardiac energy metabolism is crucial. In a healthy adult heart, over 95% of produced ATP is derived from mitochondrial oxidative phosphorylation, and this is predominantly by fatty acid oxidation (FAO) (9). However, a significant metabolic switch toward the less efficient anaerobic glycolytic metabolism occurs in failing hearts, which resembles the energy preference of the fetal heart (10, 11). The inefficiency in utilizing fatty acids results in the accumulation of lipid droplets, which subsequently lead to lipotoxicity and heart failure (12). In addition to lipid accumulation, failing hearts also exhibit impaired metabolic flexibility in switching between different energy substrates, including fatty acids, glucose, ketones, and amino acids (13). The lack of sufficient energy substrates due to prolonged fasting is, in fact, a known trigger for inherited cardiomyopathies (14). Taken together, both internal and external factors could affect cardiac performance and disease progression by disrupting metabolic homeostasis.

Cardiac tissues and plasma samples from patients carrying truncating *TTN* variants, which account for 15–20% of DCM populations, showed affected genes and metabolites involved in metabolic regulation when compared to DCM patients without *TTN* variants (15). This suggests a tight relationship between *TTN* variants and metabolic alterations. In addition, murine and human DCM hearts carrying a *PLN* variant showed suppressed mitochondrial fatty acid metabolism at mRNA and protein levels (16, 17). Suppressed metabolic genes and mitochondrial enzyme activities were also observed in 2D and 3D human induced pluripotent stem cell-derived cardiomyocytes harboring a mutated *PLN* gene (18). Multiple omics-based studies showed changes in metabolite levels, such as glutamine, lactate, and acylcarnitines, in DCM patients when compared to healthy individuals and patients with ischemic cardiomyopathy (19–21). Additionally, the metabolic changes correlated with the disease severity (19, 22). Therefore, metabolites involved in metabolic signaling, such as branched-chain amino acid metabolism, glycolysis, and glycolipid metabolism, have been proposed as potential biomarkers for DCM patients (23, 24). In line with these findings, clinical measurements using cardiac magnetic resonance imaging and positron emission tomography scanning also revealed an impaired oxidative metabolism and the subsequent energy starvation mode in DCM (12, 25). Additionally, DCM-related genetic variants, such as *LMNA* variants, show a direct influence on lipid metabolism (26). Besides the impaired fatty acid metabolism in DCM patients, individuals with FAO disorders also have a higher risk of developing DCM (27). These findings indicate a bi-directional association between DCM and impaired fatty acid metabolism. A recent study showed improved contractility and mitochondrial respiration in cardiomyocytes with various DCM-causing variants, including mutated *PLN*, *TNNT2*, *TTN*, *LMNA*, *TPM1*, and *LAMA2*, by enhancing serine metabolism (28). Serine is a non-essential amino acid and decreased serine availability has been shown to suppress mitochondrial FAO, glucose and glutamine metabolism (29), highlighting the tightly associated metabolic pathways and the promising metabolic-based treatment strategies in DCM.

High energy demand is required in HCM due to the associated hypercontractility (30). Unlike the decreased power cycle (duty ratio) and a lower force-holding capacity in DCM mutations when compared to the wildtype controls, which require much less ATP, HCM mutations exhibit an increased power cycle and a higher force-holding capacity, leading to a higher ATP usage (31). Therefore, alterations in metabolism show a profound impact on HCM pathogenesis. Additionally, in contrast to the decreased Ca^2+^ sensitivity in DCM, increased Ca^2+^ sensitivity and cytosolic adenosine diphosphate (ADP) levels are seen in HCM due to sarcomeric variants, resulting in metabolic changes (32). Increased cytosolic ADP increases the oxidation of two metabolic enzymes (NADH and NADPH), which decreases the capacity to attenuate mitochondrial reactive oxygen species (ROS) levels, as NADPH is necessary to detoxify ROS (30). Increased ROS subsequently impairs mitochondrial activation and contributes to HCM development (33). Additionally, reduced phosphocreatine (PCr)/ATP ratios in HCM, both with and without hypertrophy, indicate cardiac energetic impairments are present at an early stage of HCM (34). The switch from FAO to glucose consumption is seen in hypertrophied hearts, along with a decreased expression of CD36, a key lipid transporter (35, 36). Multiple omics-based studies comparing HCM patients to controls further revealed alterations of molecular signatures involved in a wide array of pathways suggesting fatty acid metabolism dysregulation, a reduction of acylcarnitines, and an accumulation of free fatty acids (37–39). A recent study using adult cultured rat cardiomyocytes also demonstrated that increased glucose consumption is necessary for synthesizing aspartate, which directly drives cardiomyocyte hypertrophy (40). Mouse hearts carrying mutated *MYH6*, one of the HCM-causal genes (41), showed decreased mitochondrial ATP hydrolysis (42). Additionally, a high prevalence of HCM is observed among patients with mitochondrial diseases, and several mutated mitochondrial genes are known to contribute to HCM development, such as *HADHB* (14, 43). Taken together, impaired mitochondrial lipid metabolism and the switch to glycolysis are important for HCM initiation and progression. Therefore, the potential of various metabolic compounds is currently being studied in HCM, such as perhexiline (44, 45), mavacamten (46, 47), omecamtiv mecarbil (46), and ROS scavengers (48–50). Their efficacy, however, is still to be determined.

Besides DCM and HCM, metabolic disturbances are also observed in ACM and RCM. By comparing the transcriptional landscapes between ACM and control human hearts, affected genes were enriched for several metabolic signaling, including mitochondrial dysfunction and oxidative phosphorylation (51). By comparing the plasma metabolomes between ACM patients and healthy individuals, affected metabolites and lipids further revealed several changed metabolic pathways, including lysine degradation, tryptophan metabolism, and the beta-oxidation of fatty acids (52). A recent paper compared transcriptional changes in RCM, ischemic heart disease, and valvular heart disease to control human hearts and showed that ATP metabolic processes were enriched by altered genes in RCM but not in the other two heart diseases (53). Combined, these findings highlighted the potential benefits of restoring a balanced metabolism in inherited cardiomyopathies.

## Shared and unique metabolic alterations between DCM and HCM

**FIGURE 1:**
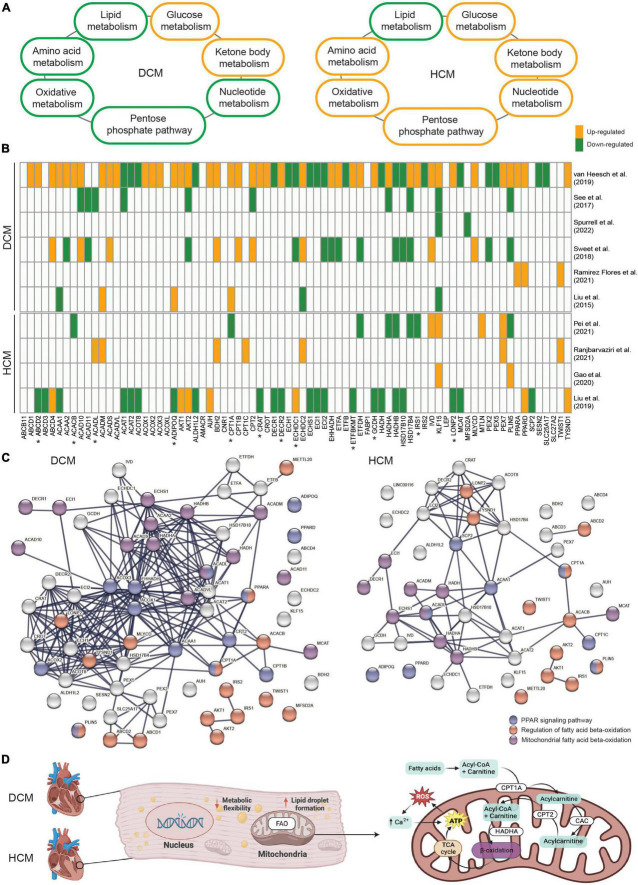
**(A)** An overview demonstrating the alteration direction of seven main metabolic processes in dilated cardiomyopathy (DCM) and hypertrophic cardiomyopathy (HCM). **(B)** The involvement and expression pattern of 77 protein-coding genes associated with fatty acid beta-oxidation (FAO) in DCM and HCM. Up-regulated genes in DCM or HCM vs. the controls are shown in orange, and down-regulated genes are shown in green. Genes that were either not significantly altered or not shown in the related paper were shown in white. Genes with opposite alteration directions between DCM and HCM are indicated by “*”. **(C)** The enriched protein-protein interaction network in DCM and HCM, respectively. Highlighted genes involved in the regulation of FAO (red), mitochondrial-related FAO (purple), and the peroxisome proliferator-activated receptor (PPAR) signaling pathway (blue). **(D)** Schematic representation of mitochondrial FAO and its key regulators in inherited cardiomyopathies.

As a result of the recent studies indicating impaired metabolism in inherited cardiomyopathies, attention has been drawn to studying and identifying precise metabolic branches and key drivers of disease pathogenesis per subtype. In general, both DCM and HCM exhibit decreased lipid metabolism (30, 54) and increased glucose metabolism (12, 55; Figure 1A). Besides these two major metabolic processes, enhanced ketone body metabolism is also shown in DCM and HCM (56, 57). Interestingly, suppressed oxidative metabolism, amino acid metabolism, pentose phosphate pathway, and nucleotide metabolism are observed in DCM (58–61), whereas they are all elevated in HCM (19, 62–65). A recent study further examined the metabolic alterations in DCM and HCM hearts as compared to non-failing control hearts at the global transcriptional level and demonstrated impaired metabolic signaling of fatty acids, carbohydrates, and amino acids in both DCM and HCM hearts (66). The study also investigated the single-nucleus transcriptome in cardiomyocytes and non-myocyte cell types and showed metabolic pathways were profoundly impaired in DCM cardiomyocytes but not HCM cardiomyocytes, suggesting the disease-specific metabolic alteration. Similarly, another paper also showed HCM- or DCM-specific impaired metabolic processes (67). Disease-specific gene sets, some of which are involved in lipid metabolism, were found to be differentially changed between DCM and HCM, such as the up-regulated *APOE* and the down-regulated *GPT* in DCM, as well as the up-regulated *APOLD1* and the down-regulated *STARD13* and *PON3* in HCM. In line with these findings, we also demonstrated that *KLF15*, an important transcription factor regulating lipid metabolism (68), was significantly up-regulated in HCM hearts carrying mutated *MYBPC3* and down-regulated in DCM hearts carrying mutated *PLN* when compared to non-failing donor hearts (16, 38). Besides the transcriptional level, proteins involved in metabolic pathways, including lipid transfer and fatty acid biosynthetic process, also showed DCM- and HCM-specific changes (69). To conclude, a profoundly impaired metabolism is well-characterized in inherited cardiomyopathies. Meanwhile, DCM- and HCM-specific metabolic alterations, particularly candidate genes in lipid metabolism, have also been cataloged.

The shared and unique metabolic signaling pathways and candidate genes have opened up new avenues to identify innovative compounds and design novel treatments for inherited cardiomyopathies in general but also for specific cardiomyopathy subtypes. For instance, mavacamten, a selective inhibitor of cardiac myosin ATPase that modulates ATP turnover time, exhibited a promising and beneficial effect on HCM patients (47, 70). FAO inhibitors and lipid-lowering agents have also been studied for treating DCM patients (71). The potential of SGLT2 inhibitors, which improve mitochondrial function, is currently under investigation in treating both DCM and HCM (72–74). Yet, the need for additional drugs targeting metabolism and mitochondrial function as the precision medicine for inherited cardiomyopathies has been urged by the Translational Committee of the Heart Failure Association and the Working Group of Myocardial Function of the European Society of Cardiology (75).

## FAO alteration in DCM and HCM

Given FAO is severely impaired in both DCM and HCM, yet a subset of FAO-related genes might be unique for each subset, we searched for relevant studies that presented global transcriptional profiles using either RNA sequencing or microarray in DCM or HCM cohorts. We filtered for studies that were conducted using either human tissue or cells or experiments that were validated in a human model after animal experiments. In total, 10 relevant papers published between 2015 and 2022 were compiled and included for the purpose of this meta-analysis. Next, we collected 76 established genes that are involved in fatty acid beta-oxidation from the gene ontology project (GO:0006635). *KLF15* was added to the gene set due to its role in FAO and its unique alteration directions in DCM and HCM. Strikingly, almost all of the 77 genes were significantly differentially expressed between DCM and control hearts in one included paper, confirming the profoundly affected lipid metabolism in DCM (Figure 1B). Interestingly, altered expression patterns for some genes, such as *IRS1* and *CPT1A*, were contrasting between DCM and HCM, suggesting the disease subtype-specific differences in the FAO impairment. Besides, *HADHA* and *HADHB*, genes coding for key enzymes in mitochondrial FAO (76), showed generally suppressed mRNA levels in DCM and HCM, whereas *ACOX1*, *ACOX2*, and *ACOX3*, genes coding for key enzymes in peroxisomal FAO (77), showed increased mRNA levels in DCM but not in HCM. This further suggests disease subtype-specific differences in subcellular organelles-related FAO impairment. It is also important to note that some FAO-related genes showed contradicting expression patterns among different DCM-based or HCM-based studies. This could be partially explained by the heterogeneous genetic variants and their mutated genes, the different disease severities of included patients, and the variable group sizes of those studies. Therefore, studies with synchronized patient cohorts and well-characterized genetic information and clinical presentations are needed to address this complex gene-disease relationship. Protein-protein interaction analysis was performed using the genes found to be differentially expressed in DCM and HCM, respectively, to further elucidate their functional networks. Among these affected genes associated with FAO, biological processes, including the regulation of FAO (GO:0031998), mitochondrial FAO (HSA-77289), and peroxisome proliferator-activated receptor (PPAR) signaling pathway (WP3942, Figure 1C), remained significantly enriched in both DCM and HCM. PPARs are important upstream transcription factors in regulating FAO and other facets of lipid metabolism and regulation (78). Notably, *PPARA* and *CPT1A* are shown in both the PPAR signaling pathway network and the regulation of FAO network. Both *ACADM* and *ACADL* overlap with the PPAR signaling pathway network and are involved with mitochondrial FAO.

## PPARA-related FAO modulators as novel candidates for the treatment of inherited cardiomyopathies?

PPARA, PPARD, and PPARG are three isoforms of the peroxisome proliferator-activated receptors (PPARs), which are ligand-activated transcription factors. Since their DNA binding regions are highly similar, they show overlapping biological functions, especially in lipid metabolism (79). PPARA is a key regulator in modulating fatty acid uptake and FAO; PPARD enhances the utilization of lipids and glucose; and PPARY increases fatty acid uptake, triglyceride formation, and lipid storage (80). Notably, multiple studies from us and others have shown suppressed PPARA expressions in DCM and HCM hearts carrying different genetic variants (16, 81, 82). Additionally, the interaction between PPARA and KLF15 showed a significant impact on cardiac lipid metabolism (83).

The cardioprotective effects of ligand-activated PPARA have been reported, including the restored balance between fatty acid uptake and FAO, increased insulin sensitivity, reduced ROS production, and attenuated fibrosis formation (80, 84). Both natural ligands (i.e., omega-3 fatty acids) and synthetic ligands (i.e., fibrates), referred to as PPARA agonists (80), are commonly used to activate PPARA. Previous studies have summarized well-established PPARA agonists and those that are still in development (85–88), some of which are PPARA-specific. Several agonists, such as bezafibrate, ciprofibrate, clofibrate, and fenofibrate, have been FDA-approved for treating type 2 diabetes or dyslipidemia, and many more are under active research (80, 87). However, most research is focused on the application of fibrates as treatments for diseases such as primary biliary cholangitis, COVID-19, and non-alcoholic fatty liver disease (89–91), and limited studies have evaluated fibrates in inherited cardiomyopathies. A recent study using knockout-*Dsg2* ACM murine hearts showed that improved myocardial fibrosis was observed after the activation of PPARA by either fenofibrate treatment or adeno-associated virus injections of PPARA (92). Nevertheless, several clinical trials have investigated the effects of bezafibrate on mitochondrial disease, neutral lipid storage disease, muscle/mitochondrial FAO disorders, and Barth syndrome (93–99), which reflect impaired mitochondrial function, lipid accumulation, and heart failure as seen in cardiomyopathies. Therefore, the results obtained from these conducted trials might also shield light on its effect on inherited cardiomyopathies.

## Conclusion

Metabolic homeostasis plays an important role in cardiac performance and disrupted metabolism is generally present in inherited cardiomyopathies, regardless of the pathogenic DNA variant and the phenotypes. Despite the shared metabolism alterations among different subtypes of inherited cardiomyopathies, etiology- and phenotype-specific metabolic impairments have been revealed, particularly in relation to FAO (Figure 1D). Those shared and unique metabolic changes provide promising candidate targets for future therapeutic strategies in treating inherited cardiomyopathies. Moreover, due to the importance of PPARA in regulating FAO and the beneficial effects of PPARA agonists observed in cardiomyocytes (100–103), studies have started to specify the pharmacological activities and cardiotoxicity of PPARA agonists (104). However, currently, there is no systematic study on the use of PPARA agonists, even FDA-approved PPARA-targeting fibrates, in patients with inherited cardiomyopathies. In conclusion, the potential of PPARA-activating drugs as FAO modulators to restore a balanced metabolism is worthy of investigation in inherited cardiomyopathies.

## Author contributions

KG-H, AB, and JP wrote the manuscript. KG-H, AB, MW, JP, and MH collected and interpreted public datasets. FWA, FvS, and MH edited the text and provided the critical input. All authors contributed to the article and approved the submitted version.
